# Clinical Outcomes and Safety of Partial Full-Thickness Myotomy versus Circular Muscle Myotomy in Peroral Endoscopic Myotomy for Achalasia Patients

**DOI:** 10.1155/2017/2676513

**Published:** 2017-02-21

**Authors:** Chenyu Li, Aixia Gong, Jingwen Zhang, Zhijun Duan, Linmei Ge, Nan Xia, Jing Leng, Mei Li, Yanjie Liu

**Affiliations:** ^1^Department of Digestive Endoscopy, The First Affiliated Hospital of Dalian Medical University, Dalian, Liaoning 116000, China; ^2^Department of Gastroenterology, The First Affiliated Hospital of Dalian Medical University, Dalian, Liaoning 116000, China

## Abstract

*Background.* Here we aimed to evaluate and compare the efficacy and safety between partial full-thickness myotomy and circular muscle myotomy during POEM procedure in achalasia patients.* Methods.* Clinical data of achalasia of cardia (AC) patients who underwent POEM in our center during January 2014 to January 2015 was collected (34 cases). 19 patients who received partial full-thickness myotomy were assigned to group A and 14 patients who received circular muscle myotomy were assigned to group B. The procedure-related parameters between the two groups were compared. Symptom relief rate and postprocedure manometry outcomes were compared to evaluate the efficacy. Procedure-related adverse events and complications were compared to evaluate the safety.* Results.* (1) Mean operation times were significantly shorter in group A than group B (62.42 ± 23.17* vs *87.86 ± 26.44 min, *p* < 0.01). (2) Symptom relief rate and postprocedure manometry outcomes had no statistical differences when compared between the two groups (all *p* > 0.05). (3) Comparison of procedure-related adverse events and complications had no statistical differences (all *p* > 0.05).* Conclusion.* Partial full-thickness myotomy had no significant differences in efficacy or safety with circular myotomy, but partial full-thickness myotomy significantly reduced the procedure time.

## 1. Introduction

Achalasia (AC) is a primary motility disease of the esophagus which presents with symptoms such as dysphagia, chest pain, regurgitation, and weight loss [1]. Without effective medical intervention, the symptoms tend to aggravate and are often associated with esophageal morphological changes which may lead to negative prognosis [2].

In the year 2010, Inoue carried out peroral endoscopic myotomy (POEM), a novel endoscopic procedure, which incorporated methods of endoscopic submucosal dissection (ESD) and natural orifice transluminal endoscopic surgery (NOTES) [3]. By comparing different surgical methods, to explore more effective and safer therapy, with an acceptable safety profile and excellent symptoms relief rate, POEM has been established as one of the best therapies for AC patients [4]. With the advantages of being less invasive, less costly, and shorter hospital stay, its preliminary efficacy such as short-term remission rate showed no difference with laparoscopic Heller myotomy (LHM) [5, 6]. However, there still exist some challenges which need further attention, such as the method of myotomy during the procedure. Theoretically, completeness of lower esophageal sphincter (LES) myotomy is the key for better outcomes. But gastroesophageal reflux disease (GERD) has always been a potential complication of the POEM procedure. It is reported that postprocedure gastroesophageal reflux rate of POEM can fluctuate between 16.5% and 60%, mostly higher than 30% [7]. Another concern has been the risk of perforation which may refer to full-thickness myotomy.

The POEM procedure has been operational in the First Affiliated Hospital of the Dalian Medical University since the year 2013, and the circular method was solely used. However, from January 2014 to January 2015, partial full-thickness myotomy was introduced as well. The focus of this retrospective study was to compare the efficiency and safety of the two methods.

## 2. Patients and Methods

### 2.1. Enrolling Criteria

Eckardt symptom score was used to select the study participant [8]. Patients with other organic diseases such as esophageal or upper gastric tumors (by esophagogastroduodenoscopy) were excluded. Diagnosis of AC was also based on at least one of other accessory examinations (esophageal manometry, barium esophagogram).

### 2.2. Exclusion Criteria

The exclusion criteria encompassed patients who had recently taken anticoagulant or hormonal drugs. Also, patients with severe cardiopulmonary dysfunction and impaired blood clotting function were exempted from the study. Likewise, patients with serious erosion or fibrosis in lower segment of esophagus or severe sigmoid-shaped esophagus were excluded [4, 9].

### 2.3. Grouping Situation

By the different methods of myotomy, 19 patients who received partial full-thickness myotomy were assigned to group A and 14 patients who received circular myotomy were assigned to group B.

### 2.4. Patients Characteristics

Patients characteristics are as summarized in Table 1. As showed, there were no significant differences (all *p* value > 0.05) in mean age, symptom duration, LESP (lower esophageal sphincter pressure), IRP (integrated relaxation pressure), length of LES (lower esophageal sphincter), and prior history of treatment between the two groups.

### 2.5. Instruments

The instruments used for POEM procedure include Olympus -260 main engine, esophagogastroduodenoscopy (GIF-Q260J, Olympus), ERBE VIO300 system, OFP water insufflation, UCR CO2 insufflator, transparent cap (D-201-11804), injection needle (NM-4L-1, Olympus), IT2 knife (KD-611), dual knife (KD-650L), hook knife (KD-620LR), biopsy forceps (FD-410LR), clips (HX-610-135, Olympus), and other accessories.

### 2.6. Procedures

All patients were admitted three day prior to the scheduled surgery. During those three days, the patients underwent electrocardiogram (ECG), routine blood analysis, blood electrolytes analysis, routing urine analysis, blood typing, chest X-ray examination, and general physical examination to ensure their suitability to go through the procedure. Patients were restricted to a clear liquid diet for 48 hours and kept 12 hours fasting water before the procedure. Prophylactic intravenous antibiotics were administered 30 minutes before the commencement of the procedure. To reduce the risk of aspiration and enhance clarity during surgery, suction by endoscopy was performed before trachea cannula.

**Figure 1:**
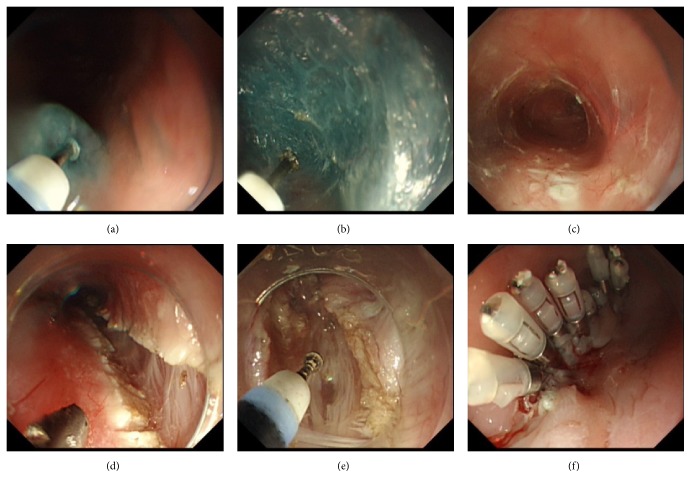
(a) Submucosal injection. (b) Creating submucosal tunnel. (c) Submucosal tunnel was dissected. (d) Circular muscle myotomy. (e) full-thickness muscle myotomy. (f) Closure of the mucosal entry site (with metallic clips).

After general anesthesia, the esophagogastric junction (EGJ) was reached with the transparent cap attached to the distal of endoscopy, and the distance from EGJ to incisors was measured by the scale along the scope. The transparent cap was used to help maintain a clear field of view [10]. Submucosal injection was then performed (at the position of 6 o'clock) approximately 10 cm proximal to EGJ (Figure 1(a)). The electrocautery knife was then used to create a 1-2 cm incision. The endoscopy was maneuvered through the incision into the submucosa (Figure 1(b)). The injection was repeated until a submucosal tunnel was created (Figure 1(c)), passing over the EGJ, for a distal tunnel of 2-3 cm into the stomach. Spindle vessels narrowing followed by widening of the submucosal tunnel at the EGJ were used as markers of entry into the gastric side [11]. The myotomy began at about 2 cm distal to the mucosal entry, approximately 8 cm above the EGJ, and the distal was extended to the fundus of the stomach in all patients.

**Figure 2:**
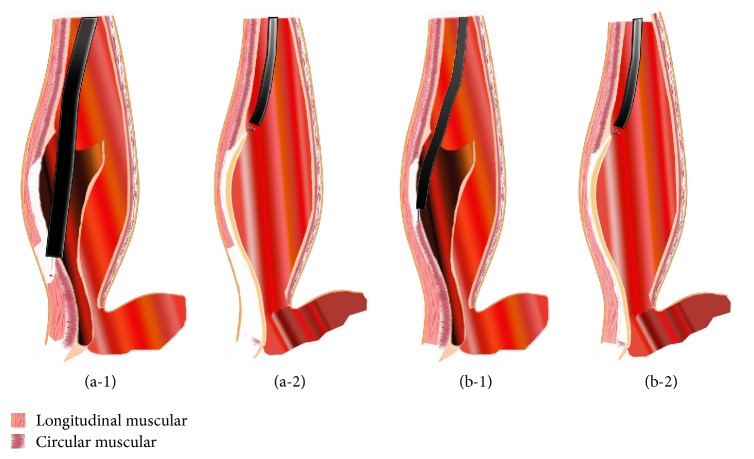
(a-1) The myotomy was begun at about 2 cm distal to the mucosal entry. In partial full-thickness myotomy, not only the circular muscle layer but also the longitudinal muscle layer was cut at 2 cm above the EGJ. (a-2) Distal of full-thickness myotomy was extended to the fundus of the stomach. Incision was closed by endoscopic metallic clips. (b-1) The myotomy was begun at about 2 cm distal to the mucosal entry in partial full-thickness myotomy. In circular muscle myotomy, only circular muscle layer was resected and the longitudinal muscle layer was carefully protected. (b-2) Distal of circular muscle myotomy was extended to the fundus of the stomach. Incision was closed by endoscopic metallic clips.

In group B, selective dissection of the circular muscular layer was done with careful protection of the longitudinal muscular layer (Figures 1(d) and 2(b-1)). In group A, a partial full-thickness myotomy was adopted. In this method, only circular muscle layer was cut from approximately 8 cm to 2 cm above the EGJ. Then both inner circular muscular layer and outer longitudinal muscular layer were cut from 2 cm above the EGJ to the fundus of the stomach (Figures 1(e) and 2(a-1)). After the myotomy was completed, the endoscopy was withdrawn and passed through the stomach to ensure that adequate increase in EGJ compliance has been achieved. The submucosal tunnel was then irrigated with antibiotic solution and the incision was closed with endoscopic metallic clips (Figures 1(f), 2(a-2), and 2(b-2)).

### 2.7. Measurements

Procedure-related parameters are operation success rate (percentage of patients who successfully received tunnel creation and myotomy), procedure time, and length of myotomy. Efficacy was compared by short-term remission rate (Eckardt score ≤ 3), recurrence rate [12], and postoperative manometry outcomes. Safety parameters are procedure-related adverse events: delayed bleeding (24 h after surgery) [13], mucosal injury, fever (temperature > 37.6°C), air related complications (subcutaneous emphysema, pneumothorax, and pneumoperitoneum), pneumonia, pleural effusion, and mediastinal effusion [14]. Clinical reflux complications are based on symptoms and endoscopic evidence (erosion).

### 2.8. Postprocedure Management

Patients were kept in supine position for 4 h. For most patients, a postoperative CT scan was performed on the first day after POEM to evaluate procedure-related adverse events such as subcutaneous emphysema, pneumothorax, and pneumoperitoneum. Patients were kept 48~72 h fasting water and on a liquid diet for an additional 1 to 2 weeks, after which soft food were introduced. All patients, postoperatively, were given prophylactic proton pump inhibitor (PPI). Oral administration was continued for at least one month after hospital discharged. Routine follow-up was undertaken for 12 months. All patients' Eckardt scores were collected (Table 2). Patients who underwent partial full-thickness myotomy were examined by manometry, upper endoscopy, and barium esophagram at 6–12 months after procedure.

### 2.9. Statistical Analysis

IBM SPSS 23.0 software was used for the statistical analysis. Measured values were expressed as means with standard deviations. Statistical significance of normally distributed data was evaluated using Student's *t*-test for independent parameters or paired *t*-test for paired samples. All reported *p* values were 2-tailed, and statistical significance was considered when *p* values were <0.05.

## 3. Results

### 3.1. Procedure-Related Parameters

The POEM procedure was accomplished successfully (tunnel creation and muscle layer myotomy) in all cases (100%). While mean tunnel creation times were similar (15.24 ± 2.81 versus 16.93 ± 3.43, *p* = 0.16), mean myotomy time and mean procedure time were significantly shorter in group A compared to group B (*p* < 0.01), as indicated in Table 3.

### 3.2. Comparison of Efficiency

The overall efficiency was notable; Eckardt score for postoperative symptom verses preoperative was 0.45 ± 0.83 versus 6.52 ± 1.82, *p* < 0.01. Postoperative Eckardt score showed no difference between the two groups (0.47 ± 0.77 versus 0.50 ± 0.94, *p* = 0.93). Treatment success rate (postoperative Eckardt score < 3 at 1st month) was 100% in both groups.

**Figure 3:**
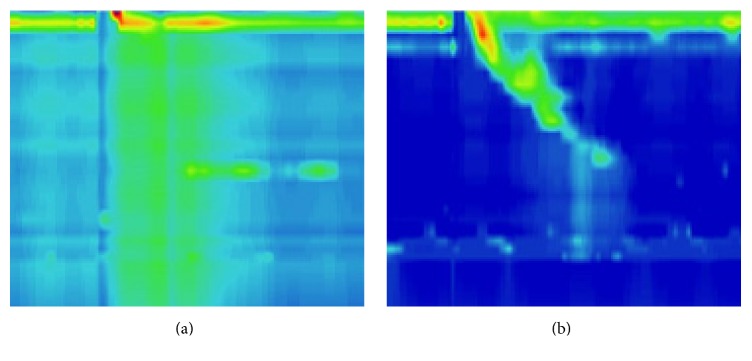
(a) A case of preoperative manometry showed Type II (absence of normal peristalsis and panoesophageal pressurization with ≥20% of swallows). (b) Postoperative manometry showed significant amelioration of panoesophageal pressurization.

Comparisons of efficiency follow-up of short-term remission rates (Eckardt score ≤ 3) in a total 33 patients at the 1st, 3rd, 6th, and 12th month were 97.0% (32 of 33), 97.0% (32 of 33), 91.1% (30 of 33), and 87.9% (29 of 33), respectively. Accomplished with manometry and gastroscopes as accessory examinations, recurrence rate was 0% in patients who underwent partial full-thickness myotomy. Remission rates in group A at 1st, 3rd, 6th, and 12th months were 100% (19 of 19), 94.7% (18 of 19), and 89.4% (17 of 19) respectively, while that in group B were 92.9% (13 of 14), 85.7% (12 of 14), and 85.7% (12 of 14), respectively. There was no significant difference between the two groups (*p* > 0.05 for all time points between the groups). Nine patients underwent postoperative manometry, 5 patients from group A and 4 patients from group B (Table 4(b)). Postoperative IRP (integrated relaxation pressure) and LESP (lower esophageal sphincter pressure) were significantly reduced compared to preoperative data (*p* < 0.01).  Figure 3 Showed the preoperative manometry and postoperative manometry micrographs of representative cases. Outcomes of postoperative IRP were similar between the two groups (3.18 ± 1.97 versus 3.05 ± 1.32, *p* = 0.91).

### 3.3. Comparison of Adverse Events

Three patients in group A and 1 patient in group B had mucosal injury (*p* = 0.62); 1 or 2 metallic clips were used for closing each of the perforations. Seven patients from group A (36.8%) and 4 patients from group B (28.6%) experienced fever on the second day after operation, while 2 patients in group A and 1 in group B developed pneumonia. No significant differences in these events were observed between the two groups. Comparisons of gas related adverse events were subcutaneous emphysema: 2 [10.5%] versus 3 [21.4%], *p* = 0.63; pneumomediastinum: 3 [15.7%] versus 2 [14.3%], *p* = 0.97; pneumoperitoneum: 3 [15.7%] versus 2 [14.3%], *p* = 0.97; and comparison of effusion were 8 [42.1%] versus 6 [42.8%], *p* = 0.96. No delayed bleeding or pneumothorax was observed (Table 5).

Detection of reflux was based on symptoms such as heartburns, regurgitation, and chest pains. During follow-up, the reflux complication rate was 10.5% (2 of 19) at 6th month and 31.5% (6 of 19) at 12th month for group A and for group B, respectively, and the corresponding incidences were 14.2% (2 of 14) and 35.7% (5 of 14), Table 6. Thus, no statistical significance existed between the groups. Ten out of the 27 (37.0%) patients who underwent postoperative esophagogastroscopy complained about reflux symptoms. Comparing group A with group B (through endoscopic findings of reflux esophagitis), the reflux rate was 26.6% (4 of 15) versus 25% (3 of 12), *p* = 0.96.

## 4. Discussion

Achalasia (AC) is an idiopathic disease of the esophagus characterized by inability of the LES to relax while swallowing. This condition weakens the quality of life of sufferers. Treatments for AC patients mostly aim at decreasing the residual pressure of the LES so that ingested material can pass into the stomach unimpeded. Previous study has showed that both endoscopic pneumatic dilation and LHM were effective in correcting the disorder [11]. Since 2010 when POEM was first applied in humans, study has confirmed its excellent outcome and advantages as compared to laparoscopic myotomy and pneumatic dilation [15]. Currently, POEM is performed in multiple centers worldwide as the first-line therapy of AC. However, some aspects or modifications such as the method of myotomy still need further improvement. Compared with circular muscle myotomy, partial full-thickness myotomy in POEM surgery also begins with selective circular muscle bundle myotomy from 2 cm distal to the mucosal entry to approximately 2 cm above the EGJ. A myotomy of both inner circular muscle layer and outer longitudinal muscle layer is then performed during partial full-thickness myotomy.

This study discussed two methods of POEM by comparing the operation success rate, procedure time, surgery efficacy, and safety. Success rates of the two groups (of the different methods) were 100% each. Mean procedure time was significantly shorter in partial full thickness myotomy group than circular muscle myotomy group. During circular muscle myotomy, much time is consumed by carefully distinguishing and protecting the longitudinal bundles. In full-thickness myotomy, however, both circular and longitudinal layers are craved in corresponding sections, and this could be the source of time saving.

As shown in Tables 4(a) and 4(b), the overall efficiency was notable. The postoperative Eckardt score significantly reduced from 6.52 ± 1.82 to 0.45 ± 0.83, *p* < 0.01, but the efficacy was equivalent between the two groups. Five patients in group A and four in group B underwent postoperative manometry, as compared with preoperative data. IRP reduced from 16.30 ± 5.05 mmH_2_O to 3.18 ± 1.97 mmH_2_O in group A (*p* = 0.04), and from 19.17 ± 1.65 mmH_2_O to 3.05 ± 1.32 mmH_2_O in group B (*p* = 0.01). It is worth noting that there was no statistical difference between the two groups with regard to postoperative esophageal pressure (*p* = 0.91), which meant that partial full thickness myotomy might not increase the incidence of postoperative reflux rate. As compared with circular muscle myotomy, partial full-thickness myotomy did not significantly reduce IRP and did not increase reflux rate.

As shown in Tables 5 and 6, there were no statistical differences in procedure-related adverse events or complications between the two groups. In total, 11 cases developed fever on the first day after the procedure. The temperature of these cases fluctuated between 37.6°C and 38.1°C. Diagnosis from routine blood test and CT revealed that two patients had aspiration pneumonia. Nine of the patients exhibited postoperative absorption heat. However, body temperatures returned to normal after the administration of intravenous antibiotic infusion combined with physical hypothermia method. Other patients with fever were asymptomatic, and no drug intervention but physical hypothermia method was used. There were three patients in group A of mucosal injury during the surgery. Two of the cases had preoperative esophagitis in lower segment. Adhesion and fibrosis due to previous esophagitis may refer to the mucosal injury during tunnel creation period. Mucosal integrity was repaired with metallic clips during the surgery.

The overall reflux complication rate by symptoms was 34.4%; the rate was 25.9% by endoscopic findings of reflux esophagitis, with no statistical difference between the two groups (all *p* > 0.05). Factors that might have accounted for these include the ingestion of acid-inhibitory drug by some patients. Some patients were exposed in acid reflux, not yet developed into esophagitis, and neurosis which was associated with patients who complained about chest pain.

Within the limitations of the study, especially the limited sample size, it is concluded that procedure time of partial full-thickness myotomy POEM was significantly shorter than circular myotomy POEM. The efficacy and safety of partial full-thickness myotomy POEM and circular myotomy POEM had no significant differences.

## Figures and Tables

**Table 1:** Preoperative patients characteristics.

Patients characteristics	Group A	Group B	*p* value
	(*n* = 19)	(*n* = 14)	
Age, y	45.37 ± 12.28	46.07 ± 15.43	0.89
Symptom duration, y	6.36 ± 7.44	5.04 ± 4.19	0.56
LESP (mmH_2_O)	27.71 ± 15.60	37.37 ± 17.41	0.19
IRP (mmH_2_O)	19.36 ± 14.10	12.60 ± 5.94	0.20
LES length (cm)	3.17 ± 1.47	3.39 ± 0.70	0.70
*Prior history of treatment*			
Heller	0 (0.0%)	0 (0.0%)	1.00
Pneumatic balloon dilation	2 (10.5%)	3 (21.4)	0.63
Botox	2 (10.5%)	1 (7.1%)	0.55
POEM	0 (0.0%)	0 (0.0%)	1.00

LESP: lower esophageal sphincter pressure, IRP: integrated relaxation pressure, LES: lower esophageal sphincter, and POEM: peroral endoscopic myotomy.

**Table 2:** Eckardt symptom score.

Score	Symptoms			
	Weight loss (Kg)	Dysphagia	Chest pain	Regurgitation
0	None	None	None	None
1	<5	Occasional	Occasional	Occasional
2	5~10	Daily	Daily	Daily
3	>10	Every meal	Every meal	Every meal

**Table 3:** Procedure-related parameters.

Parameters	Group A	Group B	*p* value
	(*n* = 19)	(*n* = 14)	
Operation success rate	100%	100%	1.00
Operation time (min)	56.73 ± 20.51	88.21 ± 27.08	**<0.01**
Tunnel creation time (min)	15.24 ± 2.81	16.93 ± 3.43	0.16
Myotomy time (min)	41.49 ± 19.71	71.29 ± 24.68	**<0.01**
Myotomy length (cm)	10.33 ± 0.90	9.86 ± 1.70	0.36

**(a):** 

Parameters	Group A	Group B	*p* value
	(*n* = 19)	(*n* = 14)	
Preoperative symptom score	6.74 ± 1.79	6.64 ± 1.86	0.89
Postoperative symptom score	0.47 ± 0.77	0.50 ± 0.94	0.93
1-month remission rate	100% (19/19)	92.9% (13/14)	0.42
6-month remission rate	94.7% (18/19)	85.7% (12/14)	0.56
12-month remission rate	89.4% (17/19)	85.7% (12/14)	0.61

**(b):** 

Parameters	Totally	Group A	Group B
	(*n* = 9)	(*n* = 5)	(*n* = 4)
IRP			
Preoperative	16.47 ± 4.89	16.30 ± 5.05	19.17 ± 1.65
Postoperative	3.12 ± 1.61	3.18 ± 1.97	3.05 ± 1.32
*p* value	**<0.01**	**0.04**	**<0.01**
LESP			
Preoperative	34.72 ± 18.58	37.60 ± 20.50	32.80 ± 10.91
Postoperative	10.49 ± 11.92	12.56 ± 15.21	9.10 ± 10.54
*p* value	**<0.01**	**0.021**	**<0.01**

IRP: integrated relaxation pressure and LESP: lower esophageal sphincter pressure.

**Table 5:** Comparison of procedure related adverse events.

Variable	Group A	Group B	*p* value
	(*n* = 19)	(*n* = 14)	
Mucosal injury	3 (15.7%)	1 (7.1%)	0.62
Fever	7 (36.8%)	4 (28.6%)	0.45
Pneumonia	2 (10.5%)	1 (7.1%)	0.55
Air-related complications	4 (21.1%)	5 (35.7%)	0.44
Subcutaneous emphysema	2 (10.5%)	3 (21.4%)	0.63
Pneumomediastinum	3 (15.7%)	2 (14.3%)	0.97
Pneumoperitoneum	3 (15.7%)	2 (14.3%)	0.97
Pneumothorax	0 (0.0%)	0 (0.0%)	1.00
Delayed bleeding	0 (0.0%)	0 (0.0%)	1.00
Effusion	8 (42.1%)	6 (42.8%)	0.96

**Table 6:** Comparison of reflux complication.

Variable	Group A	Group B	*p* value
Reflux rate by symptom at 6th	10.5% (2 of 19)	14.2% (2 of 14)	0.63
Reflux rate by symptom at 12th	31.5% (6 of 19)	35.7% (5 of 14)	0.71
Reflux rate base on endoscopy at 12th	26.7% (4 of 15)	25% (3 of 12)	0.96
